# Use of *Nigella sativa* silver nanocomposite as an alternative therapy against thioacetamide nephrotoxicity

**DOI:** 10.1186/s12263-025-00766-9

**Published:** 2025-03-14

**Authors:** Fatma M. El-Demerdash, Ansam B. Al Mhanna, Raghda A. El-Sayed, Tarek M. Mohamed, Maha M. Salem

**Affiliations:** 1 Department of Environmental Studies, Institute of Graduate Studies and Research, Alexandria University, Alexandria, 21526 Egypt; 2 Biochemistry Division, Department of Chemistry, Tanta University, Tanta, 31527 Egypt

**Keywords:** Kidney injury, *N. sativa* aqueous extract GC-MS analysis, *N. sativa-*silver nanocomposite, Thioacetamide, Oxidative stress

## Abstract

*Nigella sativa* (*N. sativa*) L. (Ranunculaceae), commonly referred to as black cumin, has a long history of usage as an herbal remedy. It has been utilized conventionally and in clinical settings to treat various illnesses. Six groups of male Wister rats were randomly selected as Gp I, represented as control; Gp II administered *N. sativa* aqueous extract (NSAE); 200 mg/kg/d, Gp III received *N. sativa* silver nanocomposite (NS-Ag-NC); 0.25 mg/kg/d; Gp IV administered thioacetamide (TAA);100 mg/kg; thrice weekly and Gps V and VI administered NSAE and NS-Ag-NC with TAA for six weeks, respectively. Findings showed that GC-MS analysis of NSAE has a high concentration of phytochemicals with strong antioxidant activity. Results revealed that TAA administration elevated TBARS, H_2_O_2_, PCC, NO levels, kidney function parameters, LDH activity, and up-regulated TNF-α, IL-1β, NF-kβ, and COX-2 gene expressions. In contrast, enzymatic and non-enzymatic antioxidants and ALP activity were extensively diminished. Also, severe abnormalities in lipid profile, hematological parameters, and histopathological features were noted. On the other hand, the administration of NSAE or NS-Ag-NC followed by TAA intoxication reduces renal impairment, restores the antioxidant system, and downregulates the expression of TNF-α, IL-1β, NF-kβ, and COX-2 genes in rats’ renal tissues. Collectively, NS-Ag-NC has more prevalent nephroprotective impacts than NSAE and can adjust the oxidant/antioxidant pathways besides their anti-inflammatory efficacy against TAA toxicity.

## Introduction

Kidneys are essential organs, responsible for controlling body functions such as erythropoiesis, calcium and phosphate metabolism, acid-base status, and intra and extracellular volume status. Chronic kidney disease (CKD) is characterized pathologically by inflammation and damage [1]. The term “CKD,” is used to describe several illnesses which affect kidney structure and function. A wide range of environmental and medical conditions might expose a person to CKD. Since CKD is a global issue that is only becoming worse, there is a rising demand for innovative preventative medicines that are efficient against diverse kidney injuries [2].

As a fungicide and a pesticide, (TAA), an organosulfur molecule, is frequently employed [3, 4]. Due to its water-soluble properties, it is a favored model toxicant. Many diseases are thought to have oxidative stress as their etiology, this is a result of reactive oxygen species [5] being produced in excess. Through the action of cytochrome P450, TAA is changed into TAA sulfoxide (TASO), and TASO is then changed into thioacetamide sulfdioxide (TASO_2_), a reactive metabolite that promotes tissue necrosis [6]. More ROS are produced because of TASO_2_ that causes increasing the production of peroxide free radicals. Therefore, oxidation processes like lipid peroxidation sped up. The kidneys are one of the organs that receive more metabolites at the end [7]. TAA significantly alters kidney function as evidenced by increased serum levels of uric acid, blood urea nitrogen (BUN), creatinine, and histological abnormalities [8].

The solubility, intestinal absorption, bioavailability, and therapeutic efficacy of drugs have been improved by the usage of nanoparticles as delivery systems [9, 10]. Significant chemical, physical, biological, and special environmental features are present in silver nanoparticles (AgNPs). Chemically synthesizing AgNPs is risky for the environment, poisonous, unstable, and expensive [11]. In contrast, one-pot biological methods for the synthesis of Ag nano-composites from medicinal plants are straightforward and can be applied instead of physical or chemical processes due to their eco-friendly, non-toxic nano-formulations, and because they don’t require the use of surfactants, co-surfactants, or capping agents [12]. Silver nanocomposites are essential for improving the bioavailability and compatibility of herbal medicines used to treat many chronic diseases, such as kidney damage.

*Nigella sativa* (*N. sativa*, NS, black seeds) has biological activities and therapeutic potential. These include antioxidant, diuretic, antihypertensive, antimicrobial, immunomodulatory, anti-inflammatory, analgesic, hepatoprotective, gastroprotective, and renal protective properties [13]. Different chemical classes have been assigned to the bioactive phytochemicals of *N. sativa*, which include major and minor secondary metabolites. The major class of *N. sativa* components was thought to be terpenes and terpenoids, such as thymoquinone (TQ) and its derivatives followed by phytosterol oil, such as β- sitosterol, Indazole alkaloids, and iso-quinoline alkaloids. Then the other minor secondary metabolites were caffeic acid, caftaric acid, gentisic acid, chlorogenic acid, p-coumaric acid, ferulic acid, sinapic acid, hyperoside, isoquercitrin, rutin, myricetin, fisetin, quercitrin, quercetin, patuletin, luteolin, and apigenin. Moreover, *N. Sativa* is an enriched natural product due to the addition of several other chemical components, including special carbohydrates, glycerolipids, phospholipids, vitamins, minerals, and some alkane hydrocarbons [14, 15]. Consequently, the goal of the current investigation is to determine the nephroprotective superiority impact of *N. sativa* silver nanocomposite (NS-Ag-NC) over the *N. sativa* aqueous extract (NSAE) against TAA toxicity by concentrating on biochemical, genotoxicity, and histopathological markers.

## Materials and methods

### Materials

*N. sativa* (NS) was provided by Imtenan Heath Shop Company, Alexandria, Egypt; thioacetamide (TAA; 98%) and silver nitrate (AgNO_3_; 99%) used in this study were acquired from Sigma-Aldrich Co. USA). The rest were analytical-grade chemicals and reagents from (Alfa Aesar Co. USA).

### Preparation of *N. sativa* seeds aqueous extract

Aqueous extract was prepared as described by Awan et al. [16]. Seeds were washed with distilled water, dried at 50 °C, and crushed in a mortar with pestle. A mixture of 10 g seed powder and 50 ml distilled water was prepared and vortexed for 15–20 min. After equilibration of 30 min, centrifugation was carried out at 1300 xg for 15 min. The supernatant was separated and filtered by Whatman^®^ filter paper no. 4. Centrifugation and filtration were repeated twice. Finally, the extract was sterilized by filtration through Acrodisc (Gelman, 0.22 μm size) and then preserved at 4 °C in a sterile bottle until use.

### GC/MS analysis

*N. sativa* aqueous seed extract was subjected to a Gas Chromatography-Mass Spectrometry (GC–MS) study using Agilent Technologies (Wilmington, Delaware, USA) equipment coupled with an HP-5MS column (30 m 0.25 mm 1D X 0.25). One microliter of the extract with a concentration of 0.5 mg/mL was injected. An ionization device with an energy of 70 eV was employed for detection. The carrier gas flow rate was kept constant at 1.1 milliliters per minute. Two hundred and fifty degrees Celsius was the injection temperature. For five minutes, the oven was set to a 60 °C isothermal warming program. After that, it warmed up by five degrees Celsius per minute for two minutes, and by ten degrees Celsius per minute for five minutes. Using the National Institute of Standards and Technology (NIST) library, the mass spectra were interpreted [17].

### Green synthesis of NS-Ag-NC

Biogenic synthesis of NS-Ag-NC from NSAE complied with the technique of Usmani et al., [18]. Briefly, 20 g of *N. sativa* seeds powder was dissolved under stirring in 400 mL of distilled water. The mixture has been heated up to boiling point for 20 min. Then, the mix was cooled and filtered. 10 mL of *N. sativa* extract was added to 0.1 mM AgNO_3_ solution then mixed for 10 min and heated for 2 h up at 80 °C and placed in the dim ambient for 24 h. The solution’s color was modified from white to pale brown and then dark brown due to the reduction of Ag^+^ and the formation of Ag nanoparticles. Further, obtained NPs were centrifuged (12,000 rpm; 10 min), and dried for 12 h at 70 °C using a vacuum oven.

### Identification of silver nanocomposite

#### FTIR evaluation

The FTIR spectrometer was managed to capture the NS-Ag-NC spectra (Shimadzu, S-8400, Japan). After the baseline was adjusted, scans were taken from 4000 to 450 cm ^− 1^ at a depth of two cm^− 1^. To be able to investigate the chemical components involved in the synthesis of NS-Ag-NC and to identify the reactive groups committed to the reduction of Ag^+^ to Ag^0^, an FTIR examination was carried out [19].

#### UV-VIS spectroscopy

After NS extraction, optical properties were assessed by UV/VIS Shimadzu Spectrophotometry (Kyoto, Japan) at 200 to 800 nm wavelength to verify the structural integrity of NS- AgNC formed [20].

### Zeta potential

Zetasizer (HT Laser, ZEN3600 Malvern Instruments, Malvern, UK) instrument was used to investigate the particle zeta potential of the prepared NS-Ag-NC [21].

### Scanning, transmission electron microscopy and energy-dispersive analysis of X-rays (EDAX)

The NS-Ag-NC were diluted with deionized water and their average size and structural features were viewed using the Scanning and Transmission Electron Microscope (Hitachi H-7650 Tokyo, Japan) [22]. Microprobe analysis of the silver nanoparticles was conducted with an energy-dispersive X-ray analysis (EDX) spectrometer. The acquisition time ranged from 60 to 100 s, and the accelerating voltage was 20 kV [23].

### Animals and experimental strategy

The animal house of the Faculty of Medicine, Alexandria University, Alexandria, Egypt, provided 42 male Wistar Albino rats, which ranged in weight from 140 to 160 g. The experimental plan was accepted locally by the Institute Animal Care Use Committee (Alexandria University local committee, Egypt) **(#AU14-220529-2-3)** and the protocol complies with the requirements of the ARRIVE Guidelines. Animals were housed in groups and provided with unlimited food and water. With 12-hour light-dark cycles, the temperature in the animal’s chamber was regulated between 21 and 24 °C. and a moisture level of 40–60%. Rats were separated into 6 groups after acclimating for two weeks. Group 1; acted as a control group and was given saline (0.4 mL/kg). Groups 2 and 3 were given NSAE (200 mg/kg) and NS-Ag-NC (0.25 mg/kg); respectively. Group 4 was administrated TAA (100 mg/kg). Groups 5 and 6 received NSAE and NS-Ag-NC one hour before the treatment with thioacetamide with the same doses mentioned above, respectively. NSAE and NS-Ag-NC were given orally once daily [24–26] while TAA was given *i.p* three times per week according to [27] for 6 weeks. Ultimately, rats from each group were euthanized after being isoflurane-anesthetized, and the kidney tissues and blood were taken for further examination.

### Hematological parameters

Complete blood picture (CBC) containing hemoglobin (Hb), red blood cells (RBCs), hematocrit, white blood cells (WBCs), platelets, and packed cell volume (PCV) were displayed from the blood samples taken using automated procedures (Sysmex kx-21n automated hematology analyzer; JAPAN CARE CO., LTD).

### Kidney functions and lipid profile

Kidney functions were evaluated by measuring the urea, creatinine, and uric acid levels using the Diamond company colorimetric kit [28–30]. Serum total cholesterol (TC) [31], triglycerides [TG] [32], and high-density lipoprotein-cholesterol (HDL-C) [33] were evaluated using commercially available kits from Bio-diagnostic, Egypt. Low-density lipoprotein-cholesterol (LDL-C) and very-low-density lipoprotein-cholesterol (VLDL-C) were also calculated. Also, alkaline phosphatase (ALP; EC 3.1.3.1) [34] and lactate dehydrogenase (LDH; EC 1.1.1.27) [35] activities were assayed in kidney homogenates.

### Oxidative stress biomarkers of kidney tissues

The concentration of thiobarbituric acid-reactive substances (TBARS) was quantified using the technique of Beheshti et al. [36]. A mixture of kidney supernatant, 1 ml TCA (20%), and 2 ml TBA (0.67%) were mixed and heated for one hour at 100 °C. Centrifugation was used to get rid of the residue after cooling. Except for the sample, a blank containing all the reagents was used to measure the sample’s absorbance at 535 nm. Hydrogen peroxide (H_2_O_2_) level was measured according to the technique of Hasan et al. [37]. Kidney samples were homogenized with trichloroacetic acid (TCA) in an ice bath. After centrifuging the homogenate at 12 000 g for 15 min, 0.5 ml of the supernatant was added to 1 ml of 1 M potassium iodide (KI) and 0.5 ml of 10 mM potassium phosphate buffer (pH 7.0). At 390 nm, the absorbance of the supernatant was measured. Protein carbonyl content (PCC), and Nitric oxide (NO) levels were measured in rats’ kidney homogenates from all groups to assess the parameters of oxidative stress [38, 39].

### Antioxidant biomarkers of kidney tissues

The activity of superoxide dismutase (SOD; EC 1.15.1.1) was determined according to Abbassy et al., [40]. The procedure involves the inhibition of epinephrine auto-oxidation in an alkaline medium (pH 10.2) to adrenochrome, which is markedly inhibited by the presence of SOD. Catalase activity (CAT; EC 1.11.1.6) was assessed spectrophotometrically at 240 nm by calculating the degradation rate of H_2_O_2_ [41], the substrate of the enzyme. Glutathione S-transferase (GST; EC 2.5.1.18) was assessed using para-nitrobenzylchloride as a substrate [42]. The activities of glutathione peroxidase (GPx; EC 1.11.1.9) and glutathione reductase (GR; EC 1.6.4.2) were measured using Hajam and Rai and Makhlouf et al. [43, 44] methods with the help of the 5,5’-dithiobis p-nitrobenzoic acid. The activity of GPx and GR was identified using a spectrophotometer to track any changes in absorbance at 412 nm. Reduced glutathione content was measured in kidney homogenates after a reaction with 5,5’- dithiobis-(2-nitrobenzoic acid) using the method of Tan et al. [45]. The yellow product 5-thio-2-nitrobenzoic acid (TNB) was measured spectrophotometrically at 412 nm. The concentration is expressed as mmol of GSH per mg of protein.

### Molecular analysis of kidney tissues

The quantitative PCR process adhered to the proposal of Kvastad et al., [46]. Table 1 presents the primer sequences. Real-time PCR was performed using Power SYBR Master Mix (Thermo Fisher Scientific, USA, K0221) on a Piko real-time thermal cycler (Thermo Scientific, Life Technology, USA). Results for the critical threshold [Ct] of the target genes were normalized to GAPDH values. Noser et al., and Livak & Schmittgen [47, 48] both previously explained how to compute the fold change in gene expression. The relative gene expression was calculated using the 2^–ΔΔCt^ method. The threshold cycle numbers of the target gene were normalized to that of the reference gene, in both the test groups and the control group by using the following equations:

**Table 1:** Primer sequences

Primer	The sequence of primer 5’-3’
IL-1β	F: GCACGATGCACCTGTACGAT
	R: CACCAAGCTTTTTTGCTGTGAGT
COX-2	F: CTGTATCCCGCCCTGCTGGTG
	R: ACTTGCGTTGATGGTGGCTGTCTT
TNF-α	F: GCCAATGGCATGGATCTCAAAG
	R: CAGAGCAATGACTCCAAAGT
NF-ĸβ	F: GCCGTGGAGTACGACAACATC
	R: TTTGAGAAGAGCTGCCAGCC
GAPDH	F: GGTGAAGGTCGGAGTCAACG
	R: TGAAGGGGTCATTGATGGCAAC

GAPDH, Glyceraldehyde-3-phosphate dehydrogenase; NF-kB, Nuclear factor kappa B; TNF-a, Tumor necrosis factor alpha; COX-2, Cyclooxigenase-2; IL-1β, Interleukins

$$\Delta Ct{\rm{ }}_{test} = Ct{\rm{ }}_{target{\rm{ }}\:in{\rm{ }}\:test\:{\rm{ }}\:groups}-Ct{\rm{ }}_{ref:{\rm{ }}\:in\:{\rm{ }}test\:groups}$$

$$\Delta Ct{\rm{ }}_{calibrator} = Ct{\rm{ }}_{target\:in\:control}-Ct_{ref: in\:control}$$

The ΔCt of the test genes were normalized to the ΔCt of the calibrator:

$$\Delta \Delta Ct = \Delta Ct_{test}-\Delta Ct_{calibrator}$$

Fold change of relative gene expression was calculated as follows:

$$Fold{\rm{ }}change = 2^{-\Delta \Delta Ct}$$

### Histopathological assessments

For the histology research, serial paraffin slices of fixed kidney tissues were made using hematoxylin and eosin stain and viewed under a light microscope. 24 h was the maximum fixing time, and the fixed tissues were preserved until they were processed in 70% ethyl alcohol. The fixed tissues were dried using a progressive series of ethanol before being embedded in paraffin [49].

### Statistical analysis

In separate experiments, each treatment’s measurements were made in triplicate. The outcomes were displayed as mean ± standard error (SE). The SPSS-22 statistical analysis program was utilized, and one-way analysis of variance (ANOVA) was employed. The pre-post” design paired-samples T-test method and post hoc multiple comparisons by Duncan multiple range test (DMRT) were used to identify the specific treatments once differences were found. The *p* < 0.05 cutoff was established as the statistical significance level.

## Results

### Identification of NSAE chemical components using GC/MS

GC/MS analysis of NSAE extract revealed the presence of thymoquinone, thymoquinol, and n-hexadecanoic acid, among other substances. Chemical components found in the NSAE along with its specifics are listed in Table 2. According to the current investigation, thymoquinone with a high concentration (14.30%) in the extract was shown to be a prominent constituent in the NSAE. Also, *N. sativa* seed yielded about 4.8% of the residue.

**Table 2:** Identified NSAE chemical components using GC/MS

Peak	Compounds	RT (Min.)	MF	MW	Percentage (%)
1	Hexanal	6.31	C_6_H_12_O	100.16	0.51
2	1-Hexanol	8.08	C_6_H_14_O	102.17	0.83
3	2-Heptanol	9.09	C_7_H_16_O	116.2	0.46
4	Dodecane	20.79	C_12_H_26_	170.33	0.60
5	Tetradecane	27.51	C_14_H_30_	198.39	0.74
6	Thymoquinone	28.81	C_10_H_12_O_2_	164.2	14.30
7	Thymoquinol	31.89	C_10_H_14_O_2_	166.22	0.64
8	n-Hexadecanoic acid	40.84	C_16_H_32_O_2_	256.42	3.21
9	(Z, Z)-9,12-Octadecadienoic acid	46.92	C_18_H_32_O_2_	280.45	3.55
10	(E)-14-Hexadecenal	52.33	C_16_H_30_O	238.41	0.82
11	n-Hexacosane	56.04	C_26_H_54_	366.70	0.91
12	n-Heptacosane	59.85	C_27_H_56_	380.73	1.30
13	Stigmastan-3,5-diene	60.97	C_29_H_48_	396.69	3.39

*MF = Molecular formula; MW = Molecular weight; RT = Retention time

### Characterization of NS-Ag-NC

**Fig. 1:**
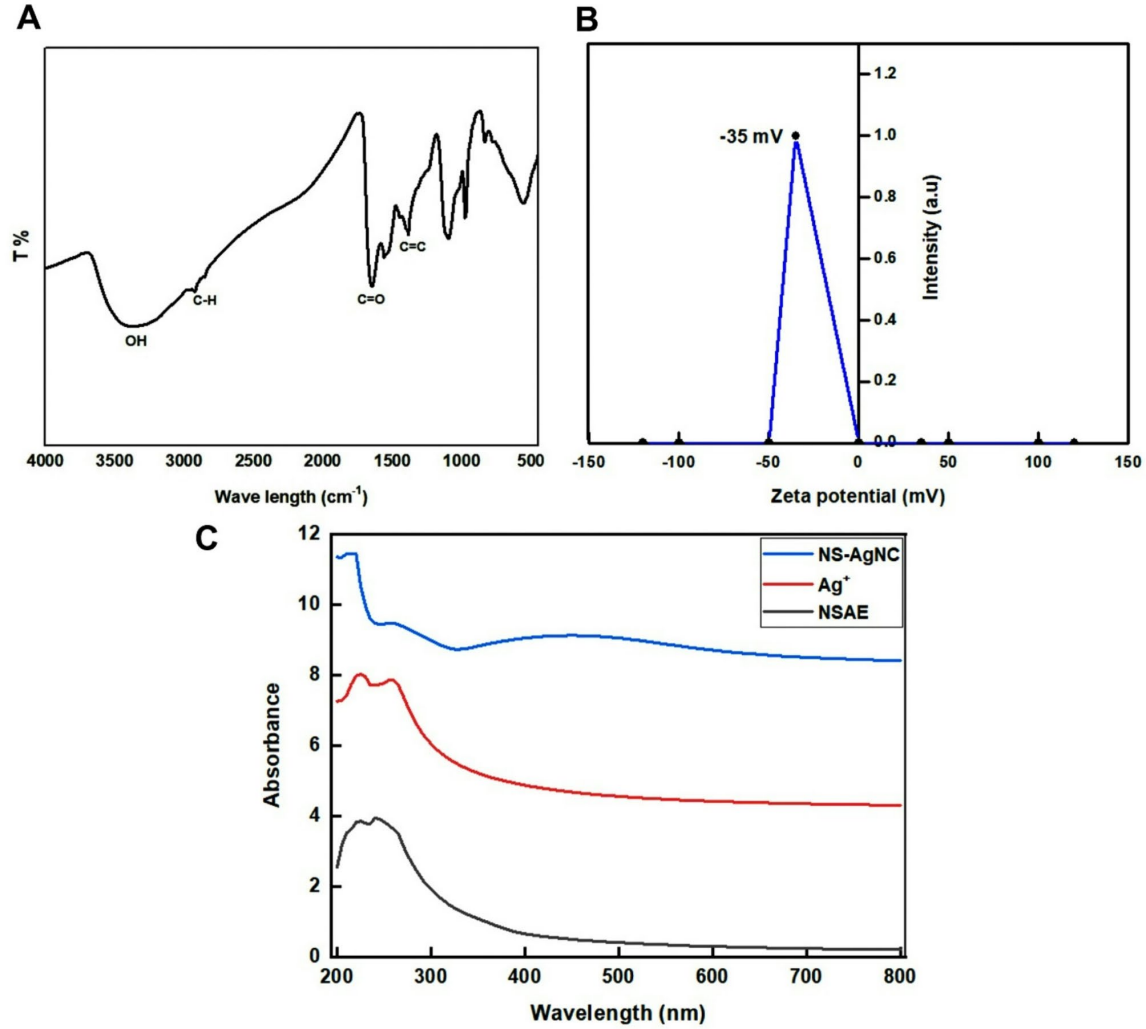
Characterization and stability of the NS-Ag-NC, **(A)** FT-IR spectra; **(B)** Zeta potential and **(C)** UV-VIS spectrum

The FT-IR spectrum showed the presence of flavonoids and polyphenols due to the presence of several absorption peaks associated with the OH of the carboxylic group, including the peak at 3351.5 cm^− 1^ that correlates to the hydrogen-bond hydroxyl and the peak at 2920.6 cm^− 1^, which demonstrated the existence of C-H. The aromatics’ C = O and C = C stretching absorption maxima were about 1647.5 and 1387.2 cm^− 1^, respectively. Additionally, several functional group peaks were visible at 975.4 and 833.4 cm^− 1^, 1367.2 cm^− 1^, 1093.7 cm^− 1^, and 554.13 cm^− 1^, which might be due to the presence of alkene, ether, alcohol, and phenols, respectively (Fig. 1A). Also, the NS-Ag-NC showed good stability of − 35 mV using zeta potential as shown in Fig. 1B. The UV-VIS absorption spectrum of NSAE, Ag^+^, and NS-Ag-NC demonstrated a characteristic band at 225, (225,255) and 450 nm respectively as shown in Fig. 1C.

**Fig. 2:**
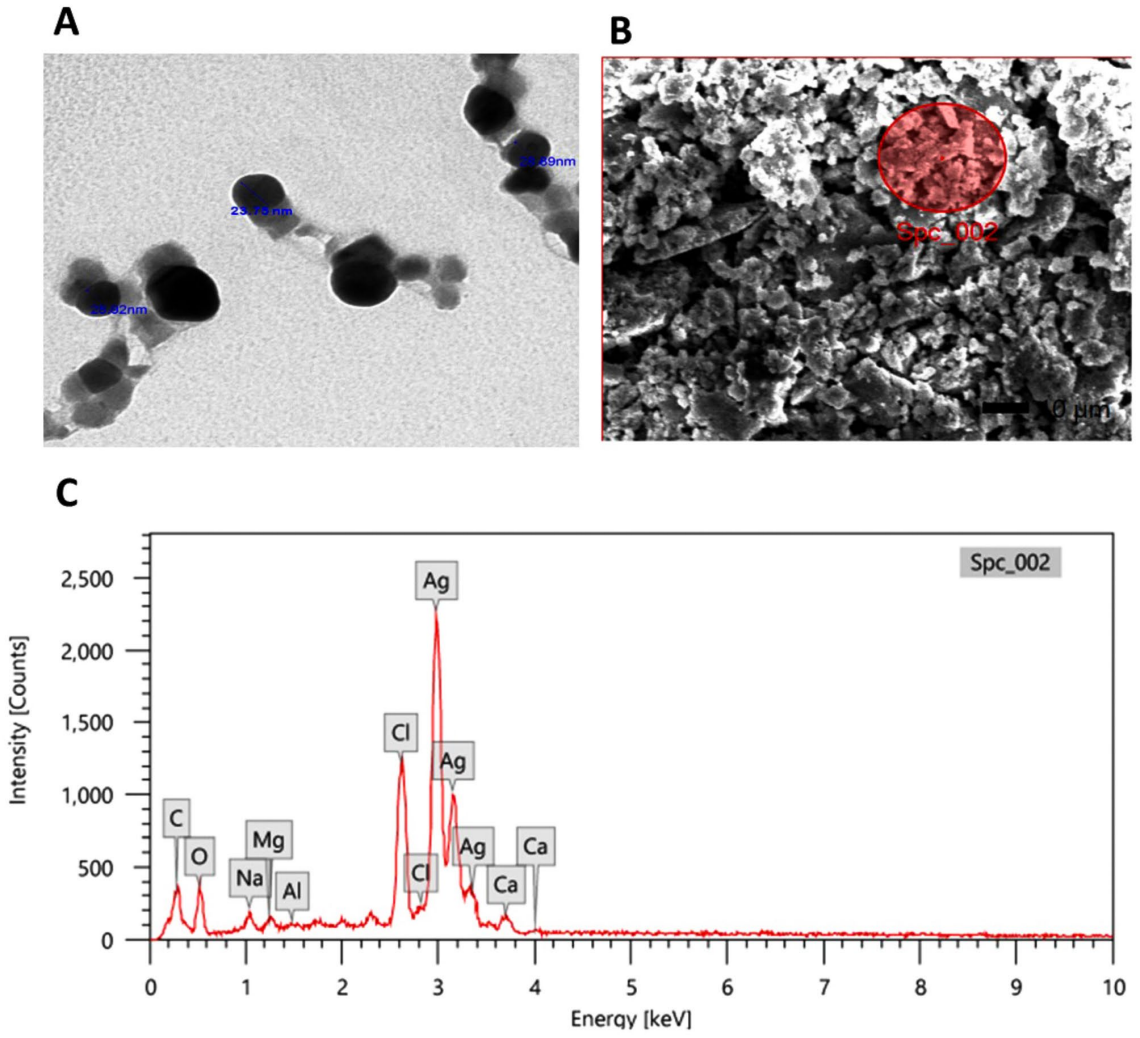
Identification of the silver nanocomposite shape and size. **(A)** Transmission electron microscopy, spherical nanoparticles at the scale of 100 nm **(B)** Scanning electron microscopy (Magnification x1,900) and **(C)** Microprobe analysis of the silver nanoparticles using an energy-dispersive X-ray analysis spectrometer

Further, the SEM and TEM analyses showed that the synthesized particles were spherical and were between 16.76 and 26.23 nm in size **(**Fig. 2A, B**)**. Another advantage of this method is the uniform dispersion and largely monodisperse nature of the particles, which eliminate the challenges posed by polydispersity in tissues. According to the previous evidence, NS-Ag-NC observed a normal relevant amount of Ag^+^ in kidney tissues equal to 0.007 µg/g using the EDX technique (Fig. 2C).

### Hematological parameters

The hematological results in Table 3 were performed on rats that had received either alone or combined dosages of TAA, NSAE, and NS-Ag-NC. Our findings revealed that WBC, platelet, and neutrophil concentrations were noticeably greater in rats given TAA in contrast to the control group by 26.65, 29.49, and 27.26%, respectively, while RBC, Hb, PCV, and lymphocyte concentrations were noticeably lower (35.19, 33.72, 21.10, 25.13; respectively). Compared to the control group, NSAE and NS-Ag-NC supplementation alone had no discernible effect on the blood parameters. Rats administrated with NSAE and NS-Ag-NC and then given TAA showed considerable restoration as compared to the TAA-treated group.

**Table 3:** Hematological parameters in different groups

Parameters	Control	NSAE	NS-Ag-NC	TAA	NSAE +TAA	NS-Ag-NC + TAA
RBCs (x 10⁶/µL)	5.70^ab^ ± 0.213	5.88 ^a^ ±0.236	5.89 ^a^ ±0.222	3.69 ^d^ ±0.099	4.72 ^c^ ±0.171	5.18 ^bc^ ±0.159
WBCs (x 10⁶/µL)	10.96^c^ ± 0.290	10.70 ^c^ ±0.38	11.23^c^ ± 0.33	13.88 ^a^ ±0.39	12.38 ^b^ ±0.37	11.40 ^c^ ±0.351
Hemoglobin (g/dl)	13.08 ^ab^ ±0.49	13.49 ^a^ ±0.29	13.67^a^ ± 0.18	8.67 ^d^ ±0.26	11.18 ^c^ ±0.33	12.21^b^ ± 0.347
Platelets (10^3^/µL)	418 ^d^ ±5.87	429 ^cd^ ±9.44	461^bcd^ ± 4.31	542 ^a^ ±18.00	474 ^b^ ±14.02	451^bc^ ± 11.43
PCV (%)	45.47 ^a^ ±1.69	44.42 ^a^ ±0.97	43.34^ab^ ± 1.38	35.88 ^c^ ±1.36	39.89 ^b^ ±1.07	43.02 ^ab^ ±1.50
Neutrophils (%)	17.18^c^ ± 0.507	17.71 ^c^ ±0.42	18.29^bc^ ± 0.57	21.86 ^a^ ±0.73	19.57 ^b^ ±0.49	18.21^bc^ ± 0.391
Lymphocytes (%)	56.29 ^a^ ±1.15	54.14 ^ab^ ±1.18	51.86^bc^ ± 0.88	42.14 ^d^ ±1.53	49.29 ^c^ ±1.46	52.57 ^abc^ ±1.49
Monocytes (%)	4.86 ^a^ ±0.173	4.66 ^a^ ±0.094	4.59 ^a^ ±0.092	4.63 ^a^ ±0.176	4.73 ^a^ ±0.127	4.68 ^a^ ±0.162

RBC: red blood cell; WBC: white blood cell; Hb: hemoglobin; PCV: packed cell volume; MCV: mean cell volume; MCH: mean corpuscular hemoglobin; MCHC: mean corpuscular hemoglobin concentration. Values are expressed as means ± SE; *n* = 7/group. Mean values within a row not sharing common superscript letters (a, b, c, d) were significantly different, (F = 20.66, *p* < 0.0001). Variations are compared as follows: NSAE, NS-Ag-NC, and TAA groups are opposed to the control group while NSAE + TAA and NS-Ag-NC + TAA groups are compared to the TAA group

### Kidney functions

**Fig. 3:**
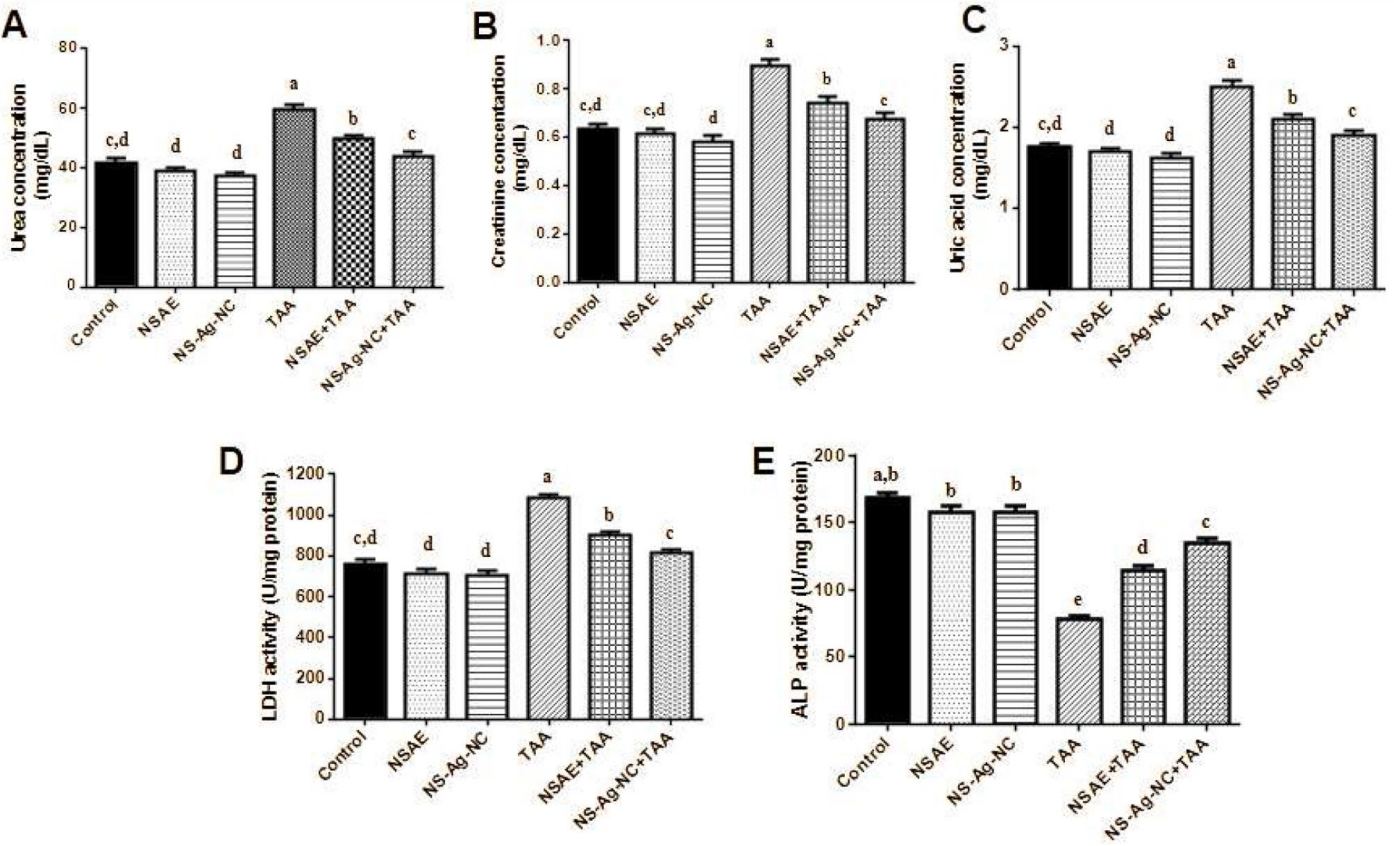
Kidney functions in serum and tissue. **(A)** Urea concentration, **(B)** Creatinine concentration, **(C)** Uric acid level, **(D)** LDH activity, and **(E)** ALP activity in the serum of male rats. Results are reported as mean ± SE of seven rats. Mean values not sharing common letters (a, b,c, d,e) were significantly different, *P* < 0.05. Variations are compared as: NSAE, NS-Ag-NC, and TAA groups are opposed to the control group while NSAE + TAA and NS-Ag-NC + TAA groups are compared to the TAA group

In this study, the renal disorder is present when serum degrees of urea (43.38%), creatinine (41.59%), and uric acid (41.98%) are increased in TAA-treated rats compared to the control group as displayed in Fig. 3. A considerable improvement was shown in serum urea, creatinine, and uric acid following the treatment of NSAE, especially NS-Ag-NC which restores renal function that had been adversely affected by TAA poisoning. Moreover, TAA administration results in a decline in ALP activity (53.63%) and an elevation in LDH activity (42.87%) in kidney tissues when compared with control rats. In contrast to TAA-intoxicated rats, receiving NSAE or NS-Ag-NC plus TAA causes a rise in ALP activity with a decline in LDH activity in rats’ kidneys with best results in the NS-Ag-NC group (Fig. 3). The administration of nanoform is more advantageous as it showed better therapeutic results in treating TAA-induced renal impairment than the standard extract (NSAE).

### Lipid profile parameters

**Fig. 4:**
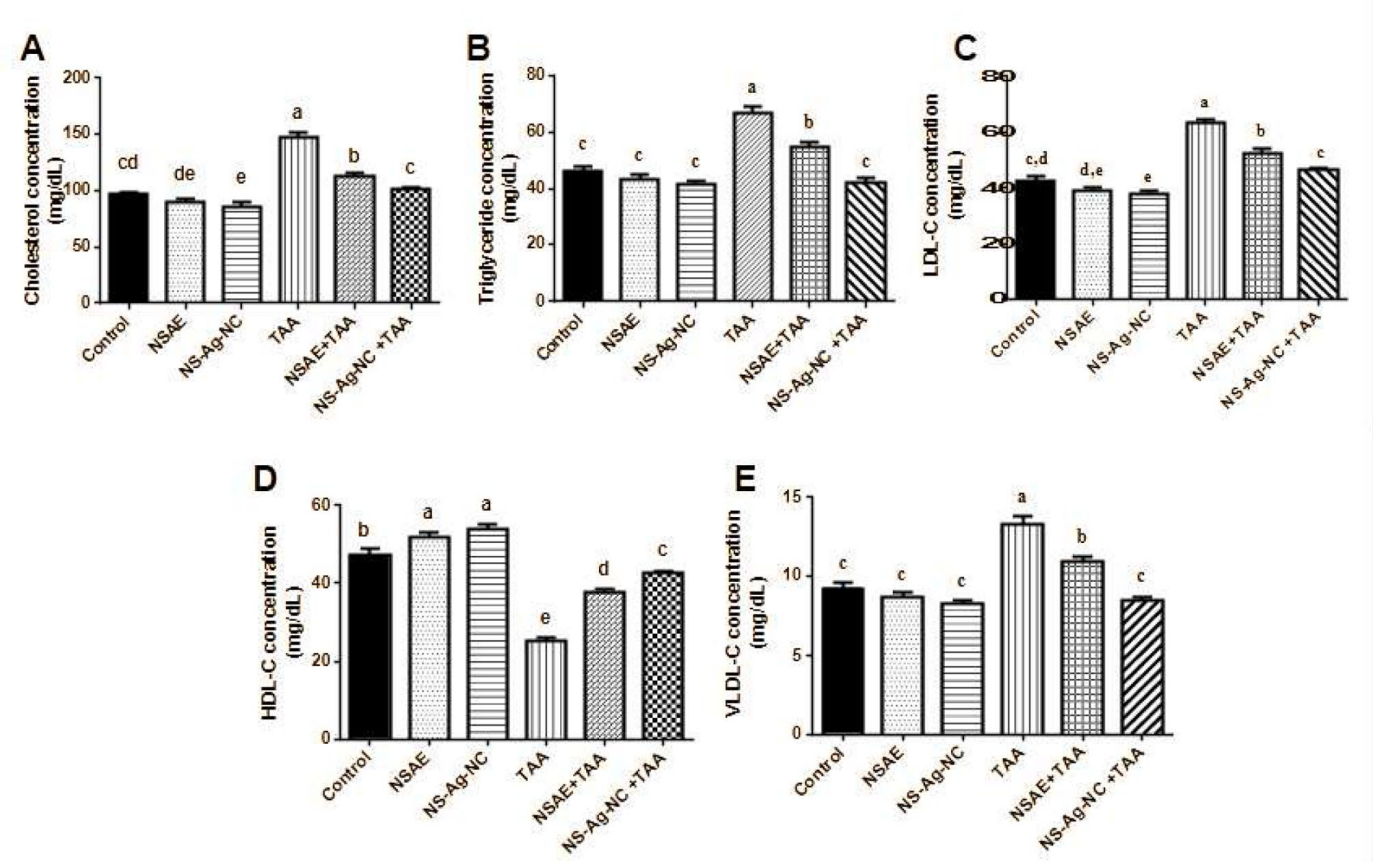
Lipid profile of rat’s serum. **(A)** Cholesterol concentration, **(B)** Triglycerides concentration, **(C)** low-density lipoprotein-cholesterol (LDL-C), **(D)** high-density lipoprotein-cholesterol (HDL-C), and **(E)** very-low-density lipoprotein-cholesterol (VLDL-C). Results are reported as mean ± SE of seven rats. Mean values not sharing common letters (a, b,c, d,e) were significantly different, *P* < 0.05. Variations are compared as follows: NSAE, NS-Ag-NC, and TAA groups are opposed to the control group while NSAE + TAA and NS-Ag-NC + TAA groups are compared to the TAA group

In comparison to the control group, total cholesterol (53.20%), LDL (48.94%), triglycerides (44.43%), and VLDL levels (44.43%) in TAA-treated rats significantly elevated, whereas HDL levels decreased by 46.47% (*p <* 0.05). On the other hand, total cholesterol, LDL, triglycerides, and VLDL levels in the NSAE and NS-Ag-NC + TAA-treated groups were substantially lesser than those in the TAA-treated rats, although HDL-C levels increased. However, treatment with NSAE and NS-Ag-NC alone significantly improved some aspects of the lipid profile in the rat serum (Fig. 4).

### Oxidative stress biomarkers in rat kidney

Changes in PCC, H_2_O_2_, NO level, and TBARS in the kidneys of male rats administered NSAE, NS-Ag-NC, TAA, or a combination of them are displayed in Table 4. According to TBARS, H_2_O_2_, PCC, and NO levels (55.16, 41.51, 43.54, and 45.20%, respectively), the oxidant/antioxidant state of TAA-treated rats was unbalanced, with noticeable increases in protein and lipid peroxidation. When contrasted to the control group, rats administered NSAE and NS-Ag-NC alone showed a remarkable (*p* < 0.05) decline in TBARS, H_2_O_2_, PCC, and NO levels. Compared to the TAA-treated group, rats treated with NSAE + TAA and NS-Ag-NC + TAA exhibited TBARS, H_2_O_2_, PCC, and NO levels close to the normal values.

**Table 4:** Oxidative stress markers in rat kidneys of different groups

Experimental groups		Parameters			
	TBARS (nmol/g tissue)	H_2_O_2_ (µmol/g tissue)	PCC (µmol/mg protein)	NO (µmol/mg protein)	
Control	26.60 ^d^ ±0.987	42.14 ^d^ ±1.40	34.77 ^d^ ±1.068	348 ^d^ ±10.57	
NSAE	21.06 ^e^ ±0.334	36.95 ^e^ ±1.08	28.70 ^e^ ±0.844	293 ^e^ ±10.15	
NS-Ag-NC	20.07 ^e^ ±0.319	34.97 ^e^ ±1.23	28.37 ^e^ ±0.947	269 ^e^ ±6.81	
TAA	41.27 ^a^ ±0.964	59.63 ^a^ ±1.35	49.90 ^a^ ±1.336	505 ^a^ ±13.19	
NSAE + TAA	33.63 ^b^ ±0.735	50.47 ^b^ ±1.55	41.85 ^b^ ±1.491	425 ^b^ ±12.45	
NS-Ag-NC + TAA	30.27 ^c^ ±0.864	46.31 ^c^ ±1.32	38.25 ^c^ ±1.137	390 ^c^ ±12.76	

Values are expressed as means ± SE; *n* = 7/group. Mean values within a column not sharing common superscript letters (a, b, c, d, e) were significantly different, (F = 113.5; *P* < 0.0001). Variations are compared as follows: NSAE, NS-Ag-NC, and TAA groups are opposed to the control group while NSAE + TAA and NS-Ag-NC + TAA groups are compared to the TAA group

### Antioxidant biomarkers in rat kidney

The kidney’s SOD, CAT, GPx, GR, and GST antioxidant enzymes as well as additional non-enzymatic antioxidants GSH are all described in detail in Table 5. Rats given TAA had significantly reduced glutathione levels (51.86%) and antioxidant enzyme activity including SOD (53.15%), CAT (51.73%), GPx (48.25%), GR (48.24%), and GST (48.64%) when contrasted to control rats (*p* < 0.05). Compared to the TAA-treated group, rats administrated NSAE + TAA and NS-Ag-NC + TAA revealed a considerable recovery in both enzymatic and nonenzymatic antioxidants. On the other hand, treatment with NSAE and NS-Ag-NC alone caused all parameters to significantly increase (*p* < 0.05) in the rat kidney.

**Table 5:** Antioxidant biomarkers in Rat’s kidneys of different groups

Groups	Parameters						
	SOD (U/mg protein)	CAT (U/mg protein)	GPx (U/mg protein)	GR (nmol/min/mg protein)	GST (µmol/h/mg protein)	GSH (mmol/mg protein)	
Control	78.23 ^b^ ±2.37	85.67 ^b^ ±1.89	58.63 ^b^ ±1.82	11.65 ^b^ ±0.405	0.678 ^b^ ±0.021	2.48 ^b^ ±0.093	
NSAE	92.37 ^a^ ±2.69	102.26^a^ ± 2.85	70.20 ^a^ ±1.64	13.65 ^a^ ±0.501	0.809 ^a^ ±0.015	3.01 ^a^ ±0.068	
NS-Ag-NC	95.17 ^a^ ±2.12	103.76^a^ ± 2.70	72.31 ^a^ ±1.80	14.13 ^a^ ±0.458	0.828 ^a^ ±0.019	3.02 ^a^ ±0.101	
TAA	36.65 ^e^ ±1.31	41.36 ^e^ ±1.58	30.34 ^e^ ±1.00	6.03 ^d^ ±0.136	0.348 ^e^ ±0.009	1.19 ^e^ ±0.027	
NSAE + TAA	57.14 ^d^ ±2.19	62.27 ^d^ ±2.25	45.93 ^d^ ±1.36	9.08 ^c^ ±0.282	0.542 ^d^ ±0.014	1.91 ^d^ ±0.041	
NS-Ag-NC + TAA	69.30 ^c^ ±2.36	73.07 ^c^ ±2.12	53.07 ^c^ ±1.25	9.91 ^c^ ±0.293	0.601^c^ ± 0.012	2.22 ^c^ ±0.077	

Values are expressed as means ± SE; *n* = 7/group. Mean values within a column not sharing common superscript letters (a, b, c, d, e) were significantly different, (F = 101.7, *P* < 0.0001). Variations are compared as follows: NSAE, NS-Ag-NC, and TAA groups are opposed to the control group while NSAE + TAA and NS-Ag-NC + TAA groups are compared to the TAA group

**Table 6:** Histopathological score of Rat’s kidneys in different groups

Groups	Hypercellular glomeruli	Dilated tubules	Rupture Bowman’s capsule and lobulated glomeruli of some renal corpuscles	Inflammation
Control	**-**	**+**	**-**	**-**
NSAE	**-**	**+**	**-**	**-**
NS-Ag-NC	**-**	**+**	**-**	**-**
TAA	**+++**	**+++**	**++**	**+++**
NSAE +TAA	**+**	**++**	**+**	**-**
NS-Ag-NC + TAA	**-**	**+**	**-**	**-**

− No histopathologic change; + Histopathology in < 20% of fields; ++ Histopathology in 20 to 60% of fields; +++ Histopathology in > 60% of fields

### RT-PCR investigations

**Fig. 5:**
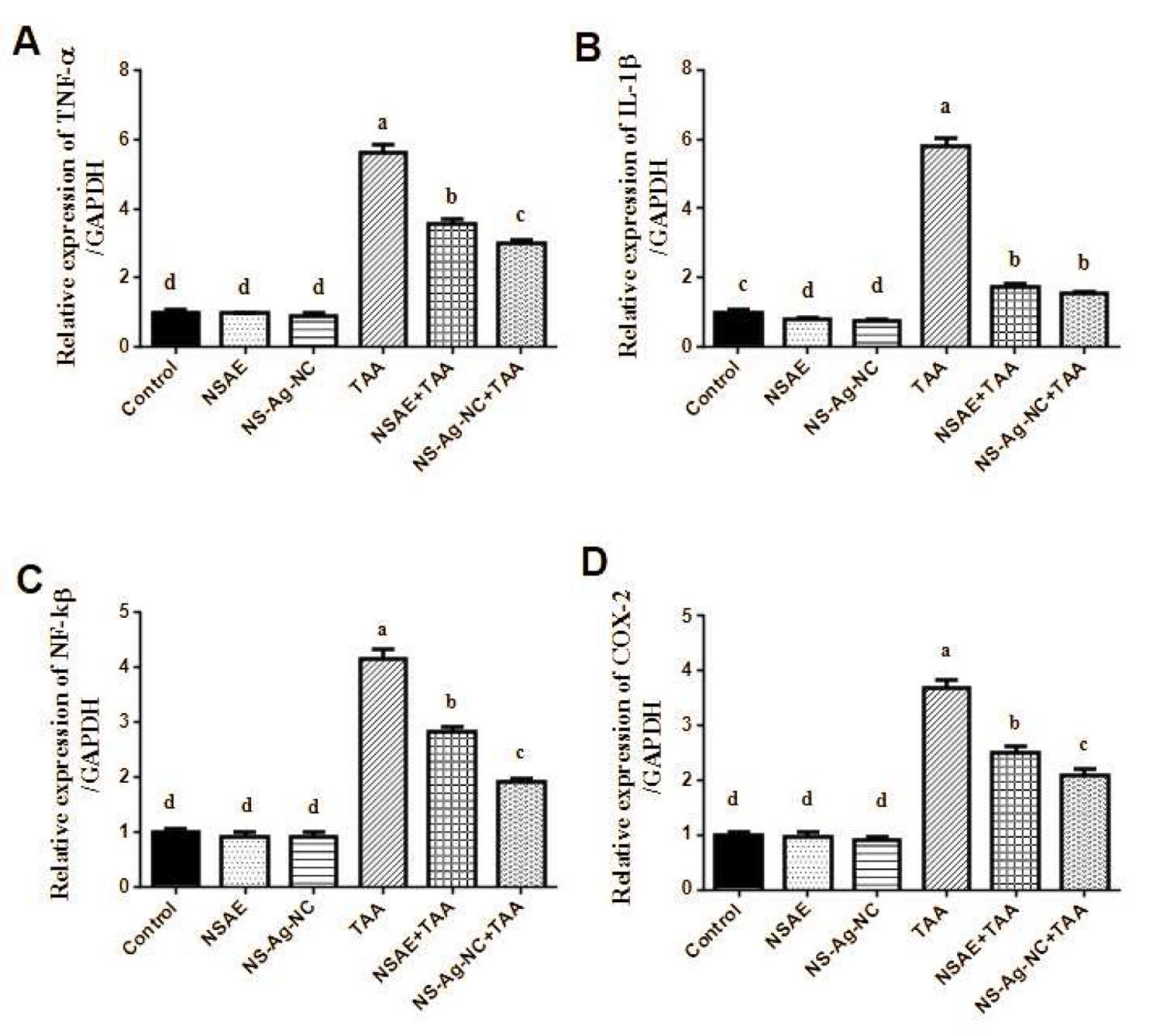
Real-time quantitative PCR analysis. **(A)** TNF-α, **(B)** IL-1β, **(C)** NF- κβ, and **(D)** COX-2 genes expression in rats kidney tissue. Results are reported as mean ± SE of seven rats. Mean values not sharing common letters (a, b,c, d) were significantly different, *P* < 0.05. Variations are compared as follows: NSAE, NS-Ag-NC, and TAA groups are opposed to the control group while NSAE + TAA and NS-Ag-NC + TAA groups are compared to the TAA group

The attained qPCR results demonstrated a substantial (*p* < 0.05) upregulation of NF-kβ, TNF-α, IL-1β, and COX-2 gene expression levels in the kidney tissues of rats treated with TAA contrasted with the normal control group (Fig. 5). The lowest expression of the NF-kβ, TNF-α, IL-1β, and COX-2 genes in the kidneys was observed after receiving NSAE and NS-Ag-NC treatments in combination with TAA. Additionally, rats administrated with NSAE or NS-Ag-NC independently showed no appreciable variation in the expression of the NF-kβ, TNF-α, IL-1β, and COX-2 genes.

### Histological examination of rat’s kidney

**Fig. 6:**
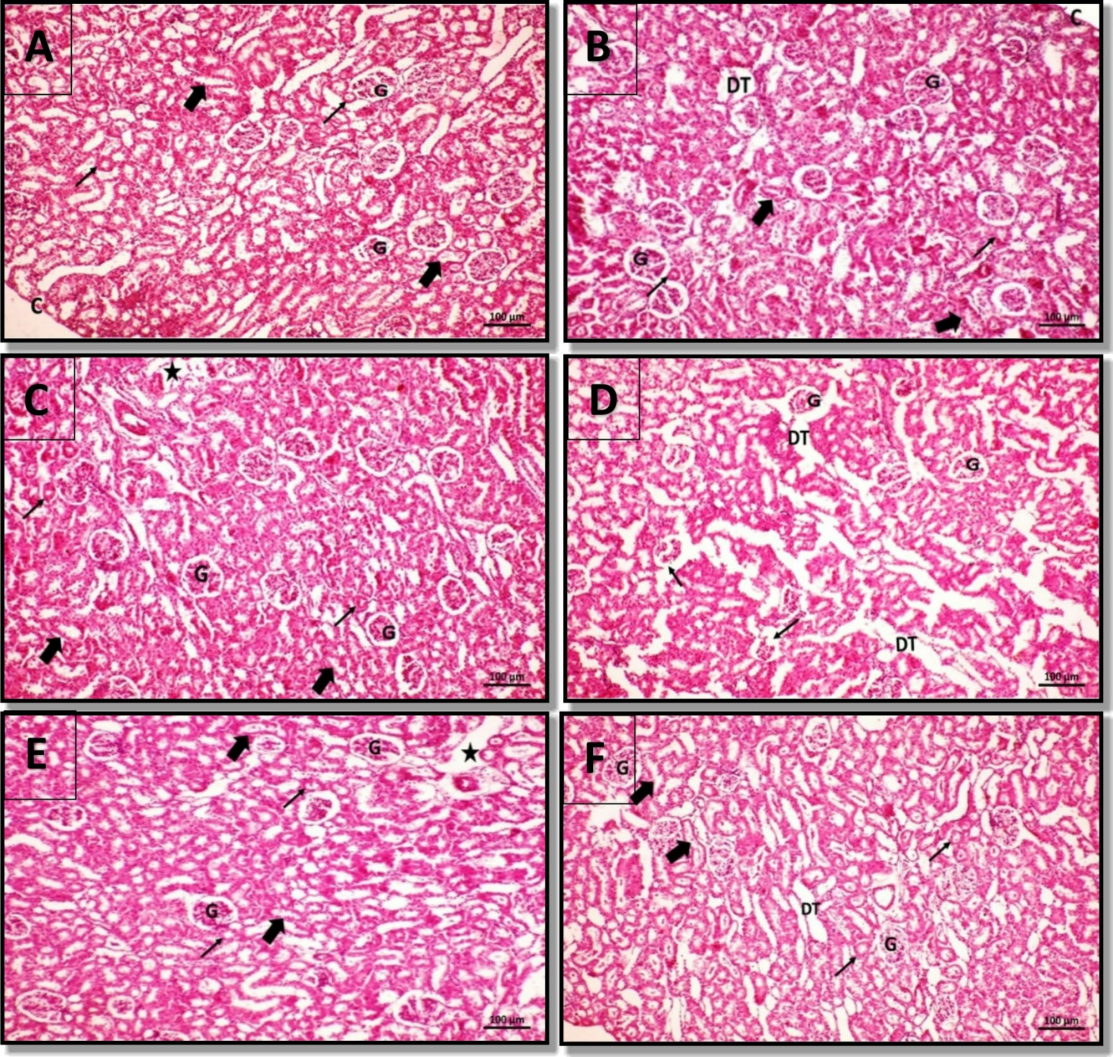
A photomicrograph of a kidney section showing **(A)** control group I with normal histological architecture. **(B)** NSAE group III with slightly normal histological architecture except for focal dilatation of renal tubules (DT). **(C)** NS-Ag-NC group IV with slightly normal histological architecture except for the widening of interstitial space at certain sites (*). **(D)** TAA group V with rupture Bowman’s capsule and lobulated glomeruli of some renal corpuscles (thin arrows) and dilatation of renal tubules (DT). **(E)** Combined NSAE and TAA-treated group VI with the normality of most renal cortical tissue except for the widening of interstitial space at certain sites (*). **(F)** Combined Ns-Ag-NC and TAA-treated group VII with normal histological architecture more or less similar to the control group except for dilatation of a few renal tubules (DT). (H&E Mic. Mag. x 200). N.B: Intact capsule (C), renal corpuscles containing glomerular tuft of capillaries **(G)**, proximal convoluted tubules PCT (thin arrows), and distal convoluted tubules DCT (thick arrows)

Histological examination of the rat’s kidney revealed that the control group showed normal histological architecture, intact capsule (C), renal corpuscles containing a glomerular tuft of capillaries (G), proximal convoluted tubules (PCT) (thin arrows) and distal convoluted tubules (DCT) (thick arrows) (Fig. 6A). NSAE group showing slightly normal histological architecture, intact capsule (C), renal corpuscles containing glomerular tuft of capillaries (G), proximal convoluted tubules (PCT) (thin arrows) and distal convoluted tubules (DCT) (thick arrows) except for focal dilatation of renal tubules (DT) (Fig. 6B). NS-Ag-NC group showing slightly normal histological architecture, renal corpuscles containing a glomerular tuft of capillaries (G), proximal convoluted tubules (PCT) (thin arrows), and distal convoluted tubules (DCT) (thick arrows) except for widening of interstitial space at certain sites (*) (Fig. 6C). While the TAA group showed marked disturbance of histological architecture, few renal corpuscles were normal containing glomerular capillaries (G), others showed rupture of Bowman’s capsule and lobulated glomeruli (thin arrows) and dilatation of PCT and DCT (DT) (Fig. 6D). In contrast, NSAE and TAA-treated groups showed improvement in histological architecture, normality of most renal corpuscles containing glomerular capillaries (G), normal proximal convoluted tubules (PCT) (thin arrows), and distal convoluted tubules (DCT) (thick arrows) except for widening of interstitial space at certain sites (*) (Fig. 6E). Moreover, Ns-Ag-NC and TAA-treated groups showed normal histological architecture more or less similar to the control group, renal corpuscles containing glomerular capillaries (G), normality of most of the PCTs (thin arrows) and DCTs (thick arrows) except for dilatation of few renal tubules (DT) (Fig. 6F). The histopathological scoring of kidney tissue was shown in Table 6.

## Discussion

Renal injury, which is characterized by renal interstitial fibrosis and glomerulosclerosis and can develop into end-stage renal failure, is a contributing factor in chronic kidney disease. Renal injury develops because of extracellular matrix aggregation and an increase in inflammatory and growth factors. TAA is considered one of the most industrial toxins and chemicals that harm the proximal renal tubule and cause renal failure. As a result, there is a growing demand for potent antifibrotic medications that work to treat fibrosis without regard to its underlying etiology [50, 51]. According to the previous demand, the current study aims to assess TAA’s renal fibrosis, and the role of NS-Ag-NC compared with NSAE in reducing its toxicity. The results of GC-mass investigated that Thymoquinone (TQ) was the most potent content inside the *N. Sativa* extract. TQ is effective in scavenging superoxide, hydroxyl radicals, and singlet molecular oxygen. Nassar, et al., [52] ascribe thymoquinone’s high antioxidant properties to the redox features of its quinine structure and its unrestricted capacity to traverse morphological barriers. This allows for easy access to subcellular compartments and facilitates the scavenging of reactive oxygen species. Lipid peroxidation of membranes is caused by the creation of highly reactive species by nitric oxide and the displacement of iron and copper ions from proteins. Studies have demonstrated that thymoquinone significantly suppressed iron-dependent microsomal lipid peroxidation. Additionally, by shielding the plasma membrane from oxidative stress, thymoquinone reversed the dysregulation of the membrane’s lipid profile [53].

Several functional group peaks were visible in the FTIR, which may have been connected to the Ag^+^ reduction and the creation of Ag^0^ nanocomposite from the NS extract [18]. UV-Vis spectroscopy showed the presence of absorption peaks appearing at 225 for NSAE, 225 and 255 for Ag^+^, and 450 nm that corresponded to absorption spectra of NS-Ag-NC. These findings confirmed the synthesis of NS-Ag-NC nanoformulation [18]. Moreover, the stability of NS-Ag-NC was − 35mV and this indicated that the NSAE can also be utilized as a capping and reducing agent to give AgNPs with colloidal stability, this was in line with the previous study of Melk et al., [21]. Green synthesis techniques were used to make the spherical nanoparticles [54], and this is how our results came about. The generated nanoparticles were shown in the SEM and TEM images to be spherical, fine, and homogeneous in size. This data was relevant to multiple reports of plant-derived extract-mediated AgNPs [55, 56]. Ultimately, the accumulation amount of Ag^+^ in the NS-Ag-NC was performed using EDX analysis. The EDX spectrum of loaded NS-Ag-NC showed a normal relevant amount of Ag^+^ and also observed an extra peak corresponding to non-toxic materials and these results agreed with Zinicovscaia et al., [57].

Blood is a liquid connective tissue that is the first component of the body to be impacted by an outside substance when it is injected, making it an accurate and sensitive indicator for determining the level of biochemical stress [58]. In this study, the erythrocytes subjected to TAA underwent morphological changes, including the loss of their unique biconcave form. At the same time, membrane protein degradation accelerated. In a prior investigation, TAA-treated rats exhibited leukocytosis, agranulocytosis, and thrombocytopenia, as well as a decline in RBC and MCV and a rise in WBC and PLT counts and these results were in line with Gheith and Abubakr and Lim et al., [59, 60].

The kidney is a crucial target organ for xenobiotics because of their wide range of damaging effects on kidney tubular cells and glomeruli [61]. The results showed that TAA administration increased amounts of urea, through the primary nitrogen-containing metabolic byproduct of protein metabolism, which prevents amino acids from being integrated into proteins [62]. In the current study, serum creatinine was significantly increased in the TAA-treated group, and the increase was connected to renal failure since serum creatinine and urea erroneously predict the glomerular filtration rate (GFR). Moreover, ALP and LDH are the main biomarkers used to evaluate kidney dysfunction and renal damage. In this work, TAA administration declined ALP and increased LDH activities in renal tissues which agreed with other findings [63, 64]. As a membrane-bound biomarker enzyme, ALP may leak into the blood consequence in tissue necrosis, which would explain why ALP levels are decreasing in kidney tissues [65]. However, LDH induction could be connected to cellular deterioration that impairs the metabolism of proteins and carbohydrates, besides energy depletion [66].

Lipids are among the biological molecules that are most susceptible to ROS. Unsaturated fatty acids, present in blood, cellular membranes, and tissues are particularly susceptible to ROS destruction. A modification in the blood’s lipid profile was discovered in earlier studies by which experimenters were subjected to heavy metals like aluminum and organophosphate insecticides [64, 67]. The observed rise in blood cholesterol levels in TAA treated group may be due to a surge in hepatic cholesterol production or it could be an indication of liver disease. due to TAA’s effects on the liver cell membrane permeability. Additionally, the obstruction of liver bile ducts may cause a decrease or cessation in the bile’s passage into the duodenum, which may be the reason why blood total cholesterol levels have increased [68]. Also, TAA treated group caused suppression of the plasma lipoproteins’ and hepatic triglycerides’ lipase enzyme activities and this caused the observed increase in serum triglycerides [69]. According to Newairy and Abdou [70], a lower level of blood HDL-C observed by TAA administration is associated with an increased risk of atherosclerosis, which is consistent with the established link between lower HDL-C levels and a higher risk of coronary artery disease [71].

Moreover, in the current study, TAA increases the formation of ROS through its metabolite, TASO_2_. Consequently, there is significant oxidative stress, lipid peroxidation, protein carbonyls and DNA adducts production, as well as other cellular harm. Additionally, ROS generates paracrine activation signals that lead to the transformation of healthy kidney cells into myofibroblast-like cells. GSH levels could be decreased because of TAA’s potential effect on glutathione production [72]. The TAA’s suppression of GR, maybe the cause of the rise in the GSSG/GSH ratio as well as the delay in GSH’s transition from an oxidized to a reduced state [73]. SOD and catalase, two antioxidant enzymes, demonstrated protection against oxidative cell damage and were inhibited in TAA treated group.

Furthermore, TAA caused secretion of TNF-α and IL-1β by Kupffer cells or neighboring sinusoidal endothelial cells, which led to severe inflammation and death, and this action was due to the consequence of lipid peroxidation and mitochondrial damage. The extracellular apoptotic signal in fibroblasts that is produced by transforming growth factor (TGF-β) is suppressed by NF-kβ upstream. Similar findings demonstrated that TAA caused structural kidney damage, alterations in trace elements, and an increase in collagen and fibrin deposition in the renal tubules and renal medulla [74]. Additionally, TAA led to cell degeneration in the proximal renal tubules’ terminal portion [75]. TAA also revealed extensive inflammatory cell infiltration of the renal tissue, glomerular sclerosis, degeneration, and necrosis, interstitial fibrosis, dilated tubules with necrotic tubular cells, and epithelial shedding [76, 77].

On the other hand, *N. sativa* is a popular remedial and natural plant that possesses a broad range of therapeutic qualities, including nephroprotective, anti-inflammatory, and antioxidant activities because it is made up of stable and volatile oils with high quantities of eicosanoids, arachidonic, and unsaturated fatty acids. The main active component is thymoquinone (TQ), carotenoids, retinol, and vitamin E [78]. The primary issue with using *N. Sativa* herbal extract during treatment is its limited bioavailability [79]. Likewise, the restricted oral bioavailability of flavonoids limits their utility as functional foods in natural medicine [80]. Poor pharmacokinetics, or low volume of distribution and strong plasma protein binding, are the main disadvantages of TQ, the main ingredient in *N. sativa* for its therapeutic uses [81]. Its bioavailability is limited by its hydrophobicity, sensitivity to light, and pH [82]. Different kinds of nanoformulations have been created recently to get over obstacles and achieve an efficient distribution [83]. Thus, the goal of the current investigation is to examine the nephroprotective superior impact of *N. sativa* -Ag nanocomposite over *N. sativa* aqueous extract against TAA renal injuries.

Interestingly, in the present study rats treated with NS-Ag-NC followed by TAA improved hematological parameters, confirming their protective effects and bioavailability with the best improvement in the NS-Ag NC and the results were following Usmani et al., and Jannathul & Lalitha [18, 84] who observed that *N. sativa -Ag nanocomposite* help occlusive vascular diseases by enhancing blood flow and hemodynamics compared with NSAE. Besides, NSAE and NS-Ag-NC administration protects the kidney damaged by TAA as shown by a considerable restoration of serum urea, creatinine, and uric acid levels. They also displayed a significant improvement in lipid profile. The best results were noted in the group that received NS-Ag-NC and TAA. Similar to our results Wells et al., [85] demonstrated that *N. sativa-*Ag nanocomposite treatment was efficient in decreasing dyslipidemia, or elevated total cholesterol or triglycerides, which may be related to its anti-inflammatory characteristics. Furthermore, Ali and Khudair [86] reported that silver nanoparticles synthesized from an aqueous extract of *N. sativa* exhibited a more pronounced ameliorative effect on lipid profiles and DNA damage than the aqueous extract of *N. sativa*. Also, NS-Ag-NC elevated ALP and declined LDH activities in kidney homogenates when compared with the TAA group which fits with a previous study [78]. Furthermore, El-Demerdash et al. [25] investigated the greater alleviated effect of *N. sativa-*Ag nanocomposite towards ALP and LDH activities.

When TAA is administered along with NS-Ag-NC, the concentration of GSH and the activity of all antioxidant enzymes such as SOD and catalase is higher contrasted with the control group, along with this effect oxidative stress was diminished due to the high concentration of bioactive polyphenolic compounds, crude oil, pure thymoquinone and Ag nanoparticles that found in NS-Ag-NC these ingredients are responsible for their excellent antioxidant’s effects against TAA toxicity. Alkhalaf et al., [24] also agreed with the ability of *N. saliva’s* antioxidant qualities to attenuate oxidative stress observed that the green synthesis of silver nanoparticles in combination with *N. sativa* extract could be a potential preventative against TAA kidney damage.

According to molecular studies, NS-Ag-NC supplementation changed the in vivo expression pattern of NF-kB, and the transcription patterns of downstream molecules including COX-2, TNF-α, and IL-1β. Consequently, the immune response and inflammation may be followed by the emergence of fibrosis throughout the healing process, and the evidence of anti-fibrotic capacity supports the study’s conclusions [87]. Additionally, Salama et al. [88] respected the renal protective effect of AgNPs combined with TQ and affirmed their anti-inflammatory effects via reducing NF-kB, TNF-α, and IL-1β biomarkers. Furthermore, Rats treated with TAA and NS-Ag-NC preserved the kidney’s histoarchitectural pattern and the nephropathic alterations brought on by TAA were improved [89]. Also, our finding was similar to Laib et al. [90] who investigated the therapeutic Efficacy of biogenic silver nanoparticles against cadmium-induced nephrotoxicity. Overall, the mechanistic insights for the previous superior mitigated effect of NS-Ag-NC was due to the antioxidant potential of silver nanoparticles (AgNPs) synthesized from *N. sativa* extracts, also their unique properties derived from both the nanoparticle targeted small size scale form and the biological activity of *N. sativa* that attributed to thymoquinone (TQ, which is a prominent component of essential oils and plays a crucial role in the formation of stable silver nanoparticles. Thus, the renoprotective capacity of NS-Ag-NC is crucial for understanding their potential health benefits and therapeutic applications.

## Conclusion

The current research indicates that TAA can alter gene expression, oxidative damage, the antioxidant defense system, and biochemical markers, all of which can lead to renal failure. Additionally, GC/MS analysis of the NSAE extract revealed the presence of important phytochemical components with considerable antioxidant activity. A potent nanoformulation, NS-Ag-NC was developed and demonstrated synergistic therapeutic efficacy in reducing nephrotoxicity caused by TAA more than NSAE. These findings highlight the prospect of *N. sativa* silver nanoparticles as a phytotherapeutic agent for TAA nephrotoxicity management. It is also advised that more studies and clinical trials be conducted to maximize its application in medical practice.

## Data Availability

The data sets used and/or analyzed during the current study are available from the corresponding authors upon reasonable request.

## Abbreviations

CKD: Chronic kidney disease
BUN: Blood urea nitrogen
GC–MS: Gas Chromatography-Mass Spectrometry
NIST: National Institute of Standards and Technology
EDX: Energy-dispersive X-ray analysis
CBC: Complete blood picture
Hb: Containing hemoglobin
RBCs: Red blood cells
WBCs: Hematocrit, white blood cells
PCV: Packed cell volume
TC: Total cholesterol
TG: Triglycerides
HDL-C: High-density lipoprotein-cholesterol
LDL-C: Lipoprotein-cholesterol
VLDL-C: Very-low-density lipoprotein-cholesterol
ALP: Alkaline phosphatase
LDH: Lactate dehydrogenase
TBARS: Thiobarbituric acid reactive substances
H_2_O_2_: Hydrogen peroxide
PCC: Protein carbonyl content
NO: Nitric oxide
SOD: Superoxide dismutase
GPx: Glutathione Peroxidase
GR: Glutathione Reductase
GST: Glutathione-S-Transferase
91: Reduced Glutathione
Ct: Critical threshold
SE: Standard error
ANOVA: One-way analysis of variance
DMRT: Duncan multiple range test
PCT: Proximal convoluted tubules
DT: Dilatation of renal tubules
DCT: Distal convoluted tubules
C: Capsule
G: Glomerular capillaries
NF-κB: Nuclear factor kappa B
TNF-α: Tumor necrosis factorα
COX-2: Cyclooxygenase − 2
IL-1β: Interleukin-1β
ROS: Reactive oxygen species
SEM: Scanning electron microscope
TEM: Transmission electron microscope
TASO: Thioacetamide sulfoxide
TASO_2_: Thioacetamide sulfdioxide
TQ: Thymoquinone
*N. sativa*, NS: *Nigella sativa*
Thioacetami: TAA
AgNO_3_: Silver nitrate
NSAE: *Nigella sativa* aqueous extract
NS-Ag-NC: *Nigella sativa* silver nanocomposite
